# Understanding why child welfare clinic attendance and growth of children in the nutrition surveillance programme is below target: lessons learnt from a mixed methods study in Ghana

**DOI:** 10.1186/s12912-018-0294-y

**Published:** 2018-06-19

**Authors:** Faith Agbozo, Esi Colecraft, Albrecht Jahn, Timothy Guetterman

**Affiliations:** 1 Department of Family and Community Health, School of Public Health, University of Health and Allied Sciences, PMG, 31 Ho, Ghana; 2 Institute of Public Health, University of Heidelberg Medical Faculty, Heidelberg, Germany; 3 Department of Nutrition and Food Science, University of Ghana, Legon, Accra, Ghana; 4 Department of Family Medicine, University of Michigan Medical School, Ann Arbor, MI USA

**Keywords:** Quantitative and qualitative research, Research methodology, Nutritional surveillance, Service utilization, Growth monitoring and promotion, Community health nursing, Child health

## Abstract

**Background:**

Growth monitoring and promotion (GMP) programmes promote not only child health but serve as a service delivery strategy to enhance coverage for other crucial nutrition-specific interventions. This study compared community-based and facility-based GMP programme with respect to attendance rates, children’s nutritional status, caregivers’ satisfaction with services received and perceptions of service providers and users on factors influencing utilization.

**Methods:**

Explanatory sequential mixed methods study conducted in Ga West municipality, Ghana. It comprised 12-month secondary data analysis using growth monitoring registers of 220 infants aged 0–3 months enrolled in two community-based (CB = 104) and two facility-based (FB = 116) child welfare clinics; cross-sectional survey (exit interview) of 232 caregiver-child pairs accessing CB (*n* = 104) and FB services (*n* = 116); and in-depth interviews with 10 health workers and 15 mothers. Quantitative data were analyzed through Fisher’s exact, unpaired t-tests, and logistic regression at 95% confidence interval (CI) using SPSS version 20. Qualitative data were analyzed by thematic content analysis using ATLAS.ti 7.0.

**Results:**

Mean annual attendance to both programmes was similar with an average of six visits per year. Only 13.6% of caregiver-child pairs attained more than nine visits in the 12-months period. At least 60% of children in both programs had improved weight-for-age z-scores (WAZ) scores during participation. Predictors for improved WAZ were being underweight at baseline (AOR:11.1, 95%CI:4.0–31.0), annual attendance of at least six visits (AOR:2.2, 95%CI:1.1–4.1) and meeting the Ghana Health Service target of nine visits (AOR:4.65, 95%CI:1.4–15.1). Compared to 31.5% CB users, significant proportion of FB caregivers (57.4%) were visited at home. Half were dissatisfied with services received (CB:55.6% vs. FB:62.0%, *p* = 0.437) citing long waiting times, negative staff attitude and extortions of money. Regarding perceptions on factors hindering service utilization, emerged themes included extremes of maternal age, high parity, postpartum socio-cultural beliefs and practices, financial commitments, undue delays, unprofessional staff behaviours, high premium on vaccination and general misconceptions about the programme.

**Conclusion:**

The association of increased attendance with improved growth reaffirms the need to strengthen primary healthcare systems to improve service delivery; sensitize caregivers on contribution of growth monitoring and promotion to early child development; and increase contacts through home visits.

## Background

Promoting child health during the window of opportunity period, that is the first thousand days starting from conception to a child’s second birthday, is crucial for survival [1]. Globally, child mortality has reduced significantly [2] from 91 deaths per 1000 live birth in 1990 to 43 in 2015 [3]. Implementation of evidenced-based cost-effective nutrition-specific and nutrition-sensitive interventions that address the immediate and underlying causes of malnutrition have contributed to this progress.

One such strategy is the growth monitoring and promotion (GMP) programme. This essential preventive health intervention uses multi-sectoral approach to routinely assess child growth. It is implemented through the health and nutrition sectors, and serves as delivery channel to achieve coverage for other nutrition-specific interventions [4]. Growth monitoring promotes early child development and is associated with long term health, economic, and social benefits [5].

This programme is widely implemented in many low resource settings at the primary healthcare level as a nutritional surveillance activity linked with health promotion [6]. The intervention involves monthly weight measurement and charting, and using the information to counsel caregivers. It creates awareness about child growth and care practices with the aim of increasing demand for other health services. The long-term goal is to serve as a focal activity in an integrated child health system where caregivers are empowered to take actions to foster growth-enabling environments for children [6].

Central to the GMP programme in Ghana are three core activities: child weighing and charting of the weight-for-age Z-scores; identification of growth faltering; and counselling of caregivers on age-appropriate infant and young child feeding. Typically, GMP services are delivered in combination with other child health services such as vaccinations, vitamin A supplementation, free distribution of insecticide-treated bed nets, birth registration, education on infection prevention and family planning motivation. GMP services are provided at child welfare clinics (CWC) predominantly by community health nurses (CHN). The clinics are run as facility-based, community-based and outreach clinics. Details of the GMP programme is documented elsewhere [7]. The facility-based CWC are static clinics situated within health facilities whereas the community-based CWC are typically mobile and outreach clinics where clinic days are scheduled.

Over the past five decades of monitoring growth in Ghana, the main challenges that have confronted the programme are unavailability of and inaccessibility to weighing centres, high participant drop outs and slow progress in reducing malnutrition rates [8]. To tackle these challenges, trained volunteers called community child growth promoters were integrated into the programme in selected community-based clinics in 2005. Their primary task includes child weighing, identification of growth faltering, vitamin A supplementation, child-centred counselling on infant and young child feeding and conducting home visitations.

Adequate participation in the programme enables caregivers to track changes in their children’s weight enabling them to associate child’s weight to overall health status [9]. It rises awareness on importance of growth charts [10] and its interpretation [11]. Moreover, optimum participation contributes to early identification of growth faltering [12], increases vaccinations coverage [13] and provides avenues for education on nutrition and health [14].

Although caregivers in Ghana are increasingly becoming aware of the importance of regular growth monitoring, [15], in the past, participation was low. Some reasons cited by caregivers in the 1990s for the low participation and high drop-out rates were advancing age of child, unavailability of and inaccessibility to the service, financial constraints, transportation difficulties, unsuitable schedules, uncomfortable venues and long waiting times [16]. In addition, service providers have cited poor knowledge of caregivers on importance of immunization, excessive workload on under-staffed health workers, and poorly motivated service providers [17] which have persisted for decades [18]. The Ghana Health Service has also attributed the irregular attendance mainly to lack of maternal interest after completion of most vaccinations [19]. Nonetheless, these challenges are not limited to Ghana. Other factors such as misconceptions about childhood malnutrition, inadequate skills of health providers, ineffective supervision and shortage of logistics have emerged in Ethiopia [15] and Zambia [9].

Evidence suggest that GMP is most effective when coverage and utilization is high [13]. As part of the nutritional surveillance system in Ghana, caregivers are encouraged to send their children aged 0–24 months old to CWCs on monthly basis to receive GMP services. In this present study, we evaluated effectiveness of the GMP programme in two community-based and two facility-based child welfare clinics in terms of two key performance indicators: utilization and undernutrition rates. We also sought to identify the factors that motivated caregivers to meet appointment schedules and their level of satisfaction with services received. Finally, we explored the perception of service providers and users on barriers to adequate participation.

## Methods

### Study location

The study was conducted in four child welfare clinics (CWCs) located in the Ga West municipality of the Greater-Accra region, Ghana. All communities within the municipality were classified into rural and urban geographic zones. Within each zone, one health facility-based and one community-based CWC was randomly selected.

### Mixed methods design

**Fig. 1:**
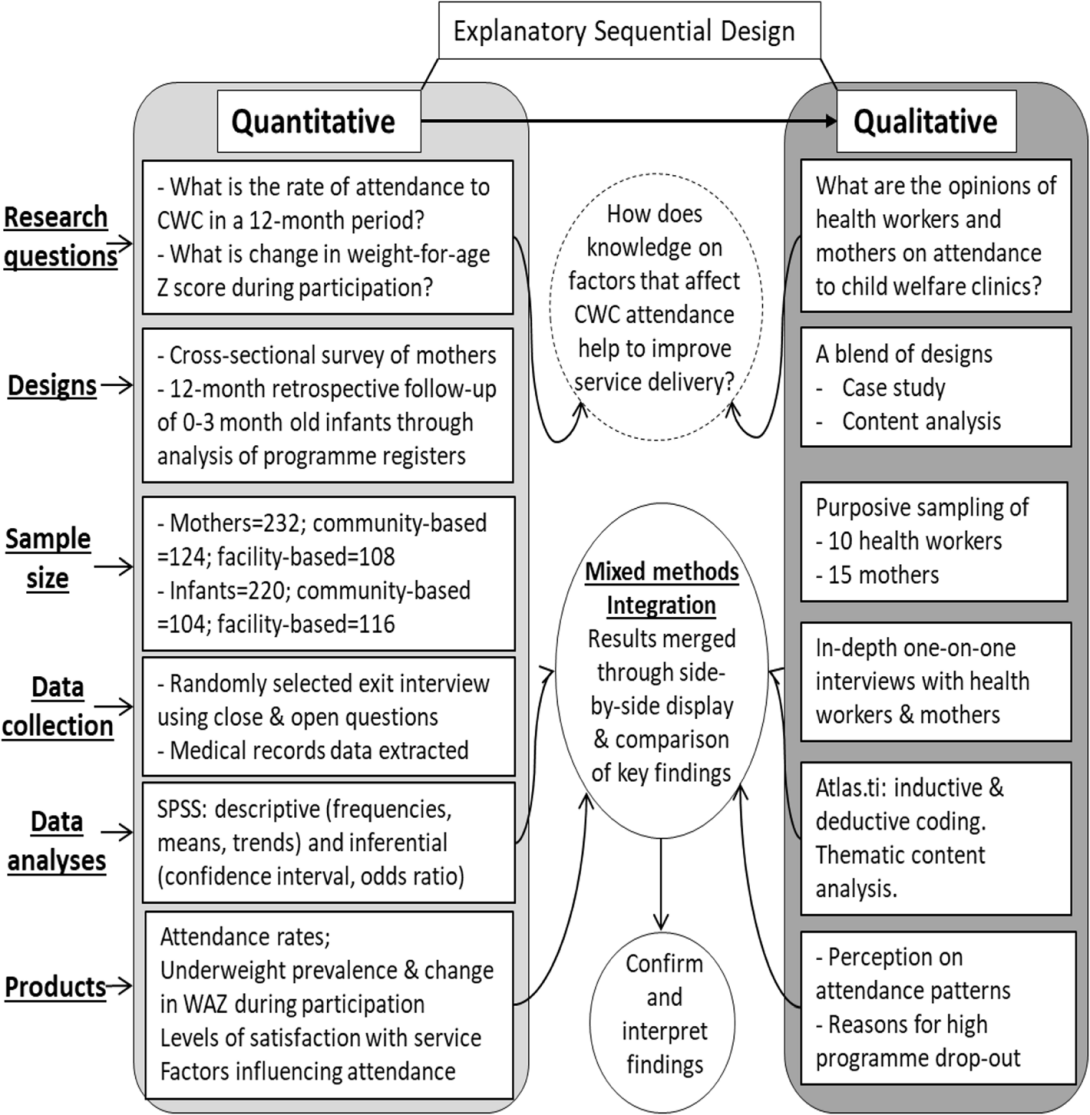
Explanatory sequential mixed methods procedural diagram

The explanatory sequential mixed methods design was used [20]. The research started with a quantitative part where the investigators assessed the levels of participation of caregiver-infant pairs enrolled on the GMP programme offered at two community-based and two facility-based CWC in 2011. The resultant change in anthropometric indices of the infants was assessed during their first and last attendance within the 12-months retrospective follow up period. Findings from the study informed the design of a qualitative follow-up in 2015. The aim was to gain in-depth understanding from service providers (community health nurses and community child growth promoters) and users (caregivers) regarding their perspectives on the attendance trends observed in the quantitative phase of the study. The step-by-step mixed methodology employed in conducting the study is shown in Fig. 1.

### Quantitative study

The quantitative part was an observational study with components of cross-sectional survey and retrospective follow-up using secondary data. The study design, data collection and analysis procedures are embedded in the mixed methods procedural diagram presented in Fig. 1.

#### Retrospective follow-up through secondary data extraction

In the closed-cohort retrospective follow-up, secondary data were extracted from the medical records over a 12-month period spanning from January to December 2011. Information on age and sex of participants, monthly attendance rates and body weight measurements were documented. The inclusion criterion was infants aged 0 to 3 months. This age bracket was chosen because of the increased likelihood of this group participating in the programme for a relatively longer period. Infants enrolled after January 2011 were excluded.

Based on the inclusion criteria, data on 220 registered infants aged 0 to 3 months was extracted from the registers of the facility-based (*n* = 116) and community-based (*n* = 104) programmes. To determine whether the sample size of 220 was sufficiently powered to generate results generalizable to the study population, post-hoc statistical power was tested. First, the standardized difference (effect size) for the two groups was calculated with the aid of a formula provided by Whitley and Ball. The 0.37 standardized difference obtained was then applied on the Altman nomogram at 0.05 alpha level. This yielded a statistical power of 83%; an indication that the sample size had the probability of detecting an effect when it actually existed [21].

A score of one was awarded for each monthly attendance and zero for non-attendance, with the maximum possible score of 12. Data on the extraction forms was entered into SPSS (version 20). Average annual visits as well as proportion of infants who attended the CWC at least six times (≥50%) or met the Ghana Health Service recommended visits of nine times (≥75%) within the 12-months period were compared using independent sample t-test and Fisher’s exact test statistic respectively. Body weight measurements were converted to weight-for-age z-scores (WAZ) using WHO AnthroPlus software (version 3.2.2). The resulting z-scores were used to determine the proportions of underweight children (WAZ < − 2 standard deviation) at baseline and at the last CWC visit within the 12-months follow-up period. Change in WAZ between first and last attendance was computed for each child and categorized as a binary variable, to indicate whether the child’s WAZ improved or deteriorated during participation. Using improvement or deterioration in WAZ as the dependent variable, a backward stepwise binary logistic regression model was built to determine the adjusted odds ratios (AOR) for improved WAZ during participation. The independent variables were type of programme, age and nutritional status of the child at enrollment, child’s sex and attendance levels.

#### Cross-sectional survey

A cross-sectional survey was conducted to assess the level of satisfaction of caregivers whose children received child welfare services in the community-based and facility-based GMP programmes. The target population was caregiver-child pairs who registered on the GMP programme between January and March 2012. To be eligible for inclusion, caregiver-infant pairs should have attended the clinic at least three times. The sample size was estimated based on a total population of 492 registered infants enrolled in the programme in the study sites at the end of December 2011. A 95% confidence level, 5% margin of error and reliability coefficient of 1.96 [22] generated a sample size of 232. This was proportionately distributed among caregivers enrolled in the community-based (*n* = 124) and facility-based (*n* = 108) programmes. A pre-tested semi-structured questionnaire with close and open-ended questions was used. The questionnaire was purposefully designed to achieve the objectives of the study. Face-to-face interviews were conducted with 232 caregivers who had received child welfare services and were exiting the clinic. Selection of caregivers to partake in the exit interviews was done randomly using the systematic sampling technique. Every fifth caregiver who assessed services and was about to leave the clinic was invited for interviewing. Brief information on socio-demography, home visitation by community health workers, and caregivers’ perceptions on the factors that motivated them to attend the clinic as scheduled were elicited. A three-point scale (satisfied, neutral and dissatisfied) was used to evaluate caregivers’ levels of satisfaction with the services received. From the pre-testing, this scale was found to be more reliable due to its robustness in clearly discriminating responses coupled with better comprehension among participants with low education. Data obtained were entered into SPSS (version 20) software and analyzed descriptively. Differences between the community-based and the facility-based programmes were determined using Fisher’s exact test and unpaired t-test reported with the corresponding 95% confidence intervals (CI) at *p* < 0.05.

### Qualitative study

A blend of case study and content analysis designs were used for the qualitative part (Fig. 1). The aim was to gain holistic understanding of the experiences of mothers who received child welfare services from facility-based and community-based CWC. The GMP programme was considered as the case whereas the content analysis provided knowledge of the phenomenon of receiving child welfare services. The case study design has been credited for its usefulness in the health sciences to evaluate programmes and interventions [23]. The qualitative content analysis facilitates a subjective interpretation of the content of text data because codes are systematically classified to identify themes [24].

#### Qualitative data collection and analysis

Ten categories of health workers and 15 mothers were purposively-selected for the qualitative follow-up. Aided by a topic guide, face-to-face in-depth interviews were conducted from July to September 2014 to explore the experiences and opinions of service providers and clients on the low patterns of attendance to the facility and community-based GMP clinics. Each interview lasted for about 45 min. Saturation was achieved after these 25 interviews necessitating termination of the qualitative data collection. The interview questions had five major domains. We delved into caregivers’ motives for taking their children to the GMP clinic, benefits derived from the programme, factors that contributed to regular or irregular attendance, effect of sporadic turnout and how participation would be improved. For instance, we asked a question on the characteristics of irregular attendees and measures to motivating caregivers to attend as scheduled.

The interview sessions were audio-recorded and afterward transcribed verbatim. The transcriptions were exported into the computer-assisted Atlas.ti software (version 7.0) for thematic content analysis. Before commencement of data analysis, a list of pre-determined codes was prepared deductively. These a priori codes were derived based on the research questions, literature on factors that influence participation in community health promotion programmes, and knowledge of the GMP programme in Ghana. Any text that could not be categorized with the initial coding scheme were given a new code. Inductive codes (in-vivo) were then generated from the textual data as result of the new ideas and concepts that emerged during analysis of the data. The lists of codes were reviewed for recurring themes. After relevant themes were mapped out from the codes, related themes were categorized into families and the thematic analysis process was completed. Important themes with accompanying quotes were extracted and summarized. Analysis was computer assisted in that the software managed the coding process and reporting.

### Mixed methods integration

The quantitative and qualitative analyses were done independent of each other. After statistical analysis of the numerical data and qualitative analysis of the textual data, key results were integrated through merging at the interpretation and reporting level. The essence of the merging was to link the quantitative to the qualitative data in order to explain findings from the latter [25]. Through comparison and visual presentation of key findings, new insights were unveiled. According to Fetters et al. [25], when similar conclusions are obtained from merged numeric and textual data, confirmation of findings provides greater credibility to the results. Presentation of quantitative and qualitative data was by side-by-side joint tabular display. Inferences and interpretations were drawn from the merged findings.

## Results

### Attendance levels and change in weight-for-age z-score

Table 1 shows attendance levels and weight-for-age z-scores (WAZ) of children in the facility- and community-based programme registers. In January when the assessment began, 32.7% (*n* = 72) of the infants were 1 mo old, 14.5% (*n* = 32) were 2 mos old, and the remainder (52.7%) were 3 mos old. Mean annual attendance was six visits (SD = 2.9). This was similar in the facility-based (5.95 ± 2.77) and community-based (6.05 ± 3.06) programmes. Overall, 46.8% (*n* = 103) of the infants received CWC services at least six times but only 13.6% (*n* = 30) met the recommended nine or more visits in the 12-months period. At baseline, mean weight of the children was 5.04 kg (SD = 1.36). Participants in the community-based programme were significantly heavier than their facility-based counterparts (5.29 ± 1.43 vs. 4.85 ± 1.28 kg). Consequently, significantly more children (19.8%) in the facility-based community-based programme were underweight compared to 8.7% in the community-based programme (Table 1). At the end of the one-year follow-up period, the overall mean weight increased to 8.87 kg (SD = 1.18). Uunderweight prevalence in the community-based cohort increased by 3%-points to 11.5% while decreasing by 4%-point to (15.5%) in the facility-based cohort (*p* = 0.436). Cumulatively, underweight levels during the 12-months period was 16.5%. Between the first and last month of attendance within the 12-months, there was about 70% increase in WAZ among both groups of children (Table 2).

**Table 1:** Comparison of anthropometric characteristics at baseline and after the 12-months observation period

Characteristics	Baseline					12th month				
	Community-based (*n* = 104)		Facility-based (*n* = 116)		*P* value	Community-based (n = 104)		Facility-based (n = 116)		*P* value
	Mean±SD	95% CI	Mean±SD	95% CI		Mean±SD	95% CI	Mean±SD	95% CI	
	n (%)		n (%)			n (%)		n (%)		
Age (months) ^a^	2.25±0.86	2.08–2.24	2.16±0.95	1.98–2.33	0.439	13.25±0.86	13.08–13.42	13.17±0.97	13.00–13.36	0.532
Weight (kg) ^a^	5.29±1.43	4.80–6.10	4.85±1.28	4.38–5.30	0.035	8.97±1.95	8.55–9.24	8.79±1.35	8.26–9.15	0.547
WAZ ^a^	− 0.60±1.04	−0.81 - -0.40	− 0.94±1.44	−1.21 - -0.71	0.049	− 0.27±1.32	−0.56 - 0.02	− 0.62±1.65	−0.94 - -0.33	0.081
Underweight (%) ^b^	9(8.7)	3.8–14.4	23(19.8)	12.9–26.7	0.022	12(11.5)	5.8–18.3	18(15.5)	9.5–22.4	0.436

^a^unpaired t test

^b^Fisher’s exact test

**Table 2:** Attendance levels and change in weight-for-age z-score during first and last appearance on the programme in the 12-months period

Characteristics	Community-based (n = 104)		Facility-based (n = 116)		*P* value
	Mean ± SD n (%)	95% CI	Mean ± SD n (%)	95% CI	
Average annual visits ^a^	5.95 ± 2.77	5.42–6.47	6.05 ± 3.06	5.48–6.62	0.800
Attend ≥6 times (%) ^b^	48 (46.2)	36.5–55.8	55 (47.4)	38.8–56.0	0.893
Attend ≥9 times (%) ^b^	13 (12.5)	5.8–18.3	17 (14.7%)	8.6–21.6	0.697
Average annual WAZ ^b^	− 0.48 ± 1.02	−0.68 - -0.29	− 0.86 ± 1.54	− 1.14 - 0.57	0.035
Change in WAZ (%) ^b^					0.772
Improved	70.2% (73)	61.5–77.9	68.1% (79)	59.5–75.9	
Deteriorated	29.8% (31)	22.1–38.5	31.9% (37)	24.1–40.5	

^a^unpaired t test

^b^Fisher’s exact test

**Fig. 2:**
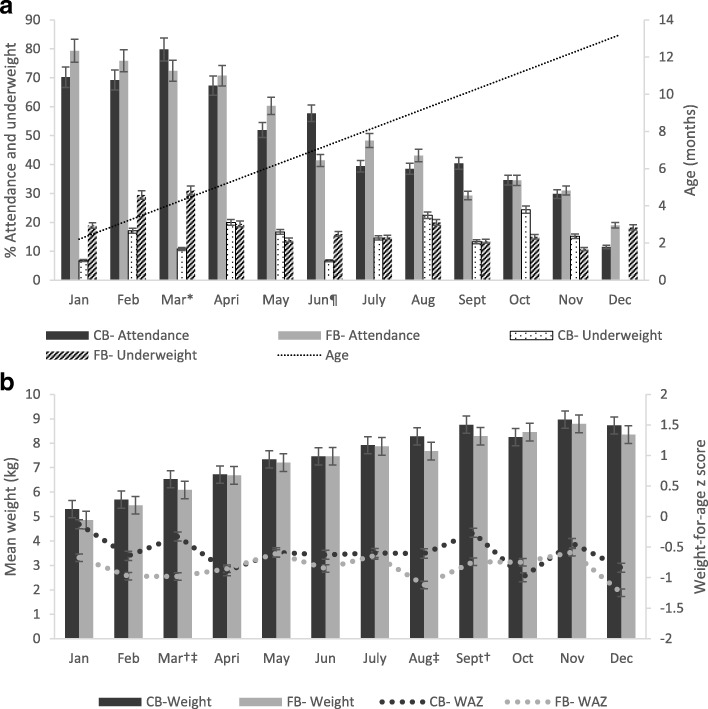
Monthly attendance and underweight prevalence per month (**a**), and the mean weight and weight-for-age z scores (**b**) in the community-based (CB) and facility-based (FB) programmes. A superscript on the month implies *p* < 0.05. Statistically significant difference for ^¶^attendance; ^*^underweight; ^†^weight-for-age z-score; and ^‡^mean weight

With regards to monthly attendance patterns, in the first month, attendance was 75%. This decreased until it tapered to the lowest rate of 16% in December 2011. Five participants (2.3%) never missed a session. The highest number of four visits was recorded by 15.5% (*n* = 34) of the participants. The overall drop-out rate was 59.5%; 58.7% in the community-based and 57.3% in the facility-based clinics. Due to the sporadic monthly attendance rates, underweight proportions within the 12-months period did not follow any peculiar trend. Underweight was highest in February (24%) and lowest in June (11%). Comparison of attendance levels, mean weight, weight-for-age z-scores and underweight prevalence in the community- and facility-based programmes is shown in Fig. 2.

### Determinants for improved WAZ during programme participation

Overall, between first and last attendance, WAZ of 69.1% children (*n* = 152, 95% CI = 63.2–75.0) improved while 30.9% (*n* = 68, 95% CI:25.0–36.8) either deteriorated (28.2%) or remained unchanged (2.7%). Infants who were underweight at baseline were eleven times more likely (95% CI:3.95–31.03, *p* < 0.0001) to increase their WAZ during participation compared to normal weight infants (Table 3). Also, females were two times more likely (95% CI:1.07–3.62, *p* = 0.029) to improve their WAZ compared to males. Children who recorded six visits (95% CI:1.14–4.07, *p* = 0.018) or met the recommended nine annual visits (95% CI:1.44–15.06, *p* = 0.010) were two and five times more likely to respectively increase their WAZ during participation. Neither enrolment in community-based or facility-based programme nor age of the child significantly affected the change in WAZ.

**Table 3:** Binary logistic regression model showing factors that predicted an increase in weight-for-age z-score between the first and last attendance on the programme

Variables	*n*	%	Adjusted regression model		
			AOR	95% CI	*P* value
Programme (Facility-based)	116	47.3	Ref		
Community-based	104	52.7	1.27	0.70–2.33	0.434
Age at registration (> 4 weeks)	148	67.3	Ref		
Neonatal (≤4 weeks)	72	32.7	0.75	0.39–1.44	0.382
Sex (male)	110	50.0	Ref		
Female	110	50.0	1.97	1.07–3.62	0.029
Underweight at baseline (No)	188	85.5	Ref		
Yes	32	14.5	11.07	3.95–31.03	< 0.0001
Annual attendance (< 6 visits)	117	53.2	Ref		
≥ 6 visits	103	46.8	2.15	1.14–4.07	0.018
Attendance (< 9 annual visits)	190	86.4	Ref		
≥ 9 annual visits	30	13.6	4.65	1.44–15.06	0.010

Hosmer-Lemeshow goodness-of-fit test, Chi-square = 10.192, *p* = 0.252. Cox and Snell R^2^ = 0.155, Nagelkerke R^2^ = 0.210

### Caregivers’ level of satisfaction with GMP services

Socio-demographic characteristics of the 232 caregivers who participated in the cross-sectional survey have been extensively described in a previous publication [7]. Mean age of the caregivers was 26 years (±5.6 SD) ranging from 15 to 45 years. Eighty-five percent (*n* = 197) lived with a partner while the remainder were single parents. One third of the mothers (37.5%, *n* = 87) were educated to the junior high school level (equivalent to the eighth grade) and 16.8% (*n* = 39) to the secondary high school/technical level (equivalent to the twelfth grade). One-fifth (17.2%, *n* = 40) had no formal education whereas only one participant had tertiary education. Almost 80% were engaged in some form of income generating venture. The mean parity among the women was three children and the average household size was five members. Mean age of the children sent to the CWC was eight months (7.9 ± 5.0 months). Although majority of caregivers registered their infants before within the neonatal period, it was significantly higher in the facility-based group (93% vs. 81%). Also, 57% of the caregivers who used the facility-based services reported being visited at home at least once by a community health worker, compared to 32% in the community-based programme (*p* < 0.0001). In both groups, caregivers mentioned knowledge on their child’s growth status and vaccinations as the most important motivating factors for regular attendance. Overall, 35% expressed satisfaction with the services received citing education on child care and the free complementary services such as birth registration and distribution of insecticide-treated bed nets. However, over half of the caregivers expressed dissatisfaction citing long waiting times, unfriendly staff attitude and service fees charged by some staff particularly the community volunteers. Breakdown of the responses in the community- and facility-based CWC is presented in Table 4.

**Table 4:** Caregivers’ experiences with the community- and facility-based child growth monitoring and promotion programmes

Variables	Community-based (*n* = 124)		Facility-based (n = 108)		*P* value
	n (%)	95% CI	n (%)	95% CI	
Enrollment time					0.047
At birth	33 (26.6)	18.5–35.5	29 (26.9)	18.5–35.2	
Two weeks after birth	36 (29.0)	21.8–37.9	43 (39.8)	29.6–50.0	
1 month after birth	31 (25.0)	17.7–33.1	28 (25.9)	18.5–35.2	
After neonatal period	24 (19.4)	12.9–25.8	8 (7.4)	2.8–13.0	
Visited at home	39 (31.5)	23.4–39.5	57.4 (62)	48.1–66.7	< 0.0001
Motivation for participation					0.004
Know child’s growth	58 (46.8)	38.7–56.5	39 (36.1)	26.9–45.4	
Receive vaccinations	38 (30.6)	22.6–38.7	19 (17.6)	11.1–25.0	
Treat minor ailments	22 (17.7)	11.3–25.0	38 (35.2)	25.9–43.5	
Nutrition education	4 (3.2)	0.8–6.5	8.3 (9)	3.7–13.9	
None	2 (1.6)	0–4.0	3 (2.8)	0.0–6.5	
Satisfied with services					0.437
Yes	43 (34.7)	25.8–42.7	29 (26.9)	5.6–16.7	
Neutral	12 (9.7)	4.8–15.3	12 (11.1)	18.5–35.2	
No	69 (55.6)	46.8–64.5	97 (62.0)	52.8–70.4	
Reasons for satisfaction					0.774
Knowledge on child’s growth	31 (70.5)	56.8–81.8	19 (65.5)	48.3–82.8	
Educated on child care	8 (18.2)	6.8–29.5	5 (17.2)	3.4–31.0	
Free complementary services	5 (11.4)	2.3–20.5	5 (17.2)	3.4–31.0	
Reasons for dissatisfaction					0.051
Long waiting times	43 (62.3)	52.2–73.9	53 (77.9)	67.6–86.8	
Unfriendly staff attitude	15 (21.7)	13.0–31.9	12 (17.6)	8.8–27.9	
Extortion of money	11 (15.9)	7.3–24.6	3 (4.4)	0–10.3	

### Characteristics of participants in the qualitative study

In-depth interviews were conducted with ten health workers who ran the GMP programme or supervised its implementation and 15 clients who accessed the service. The aim was to explore perceptions on the trends in attendance observed in the quantitative study.

Four out of the ten health workers were stationed at the district as core members of the District Health Management Team and the remainder worked at the primary healthcare level. The district health team comprised two Public Health Nurses, one nutrition officer and one disease control officer. The public health nurses had over 15 years work experience and played supervisory roles as nurse-managers. The nutrition and disease control officers provided logistics and technical support for the programme. Four were low cadre community health nurses (CHN) with on average, ten years post-qualification experience. One was selected from each of the four study facilities. The remaining two interviewees were the trained community child growth promoters who supported the CHNs and served as liaison with the community.

The mothers’ age ranged from 21 to 46 years, and the age of their accompanying children ranged from 4 to 18 months. Six mothers were uniparous and the remaining nine were multiparous with the highest parity being six children. Two had no formal education whereas three had primary education. Five completed junior (eighth grade) and senior high schools (twelfth grade). In terms of occupation, three were unemployed, eight were traders and four were self-employed artisan. Seven used community-based and eight used facility-based CWC.

### Perception of service providers/users on reasons for low attendance to CWC

The process of coding and thematic analysis of the transcribed data led to the generation of about 50 initial codes. By summarizing and synthesizing the codes, it was reorganized into 16 non-hierarchical codes from which seven themes were developed. The themes elucidated why attendance to child welfare clinics were often below set targets and the drop-out rate was high. The seven themes are described below.

#### Extremes of maternal age and high parity

It was deduced that teenage mothers and women above the age of 40 years who were grand multiparous mothers (with more than five children) failed to attend the clinics as scheduled. While the teenagers were scolded for “doing what was the reserve of their mothers”, the older and multiparous mothers were condemned for not adopting a family planning method to avert the pregnancy, despite numerous sensitization campaigns. This 43 years old mother of five children expressed her sentiment:

*“*Without considering the presence of other mothers, the nurse asked me why I gave birth at my age when I already had five children while they (the nurses) were preaching family planning every time. I felt so embarrassed”.

#### Socio-cultural beliefs and practices during early postpartum

Both the health workers and the mothers mentioned conformity to certain entrenched postpartum cultural practices as a reason for irregular CWC attendance. In certain cultures, women with newborn babies were required to remain in-doors till the umbilical cord of the newborn fell off; the circumcision wound of the baby boy completely healed or until the baby was ‘out-doored’ and/or the naming ceremony performed. Another reason that strongly emanated was the practice of mothers-newborn pairs adorning in all white attractive outfits as a way of expressing joy and gratitude to God for safe delivery. Mothers who were unable to purchase new set of clothing and apparels for adornment were reluctant to go to public places. A public health nurse at the district health directorate and a mother confirmed this assertion:

*“….*as tradition demands, newly delivered mothers wear white clothes as a way of openly expressing their happiness for safe delivery. If the mothers cannot afford to buy new clothes, especially teenage and single mothers, they are likely to remain at home. We keep telling them that the health of the infant is more important than their looks”.

“My mother told me to stay indoors for some time to keep evil eyes off my baby. She said the baby is too young to be exposed. It was very difficult convincing her to come for today’s weighing” (Mother age 22 years).

#### Financial commitments

Financial commitments towards transportation, purchasing items sold at the clinic and sometimes paying service charges was a major concern. Mothers lamented about constant persuasion from the health workers to purchase stuff they sold at the clinic. These items ranged from weighing pants, ‘weanimix’ (complementary food produced from cereal-legume-peanut blend), cosmetics, diapers to medications especially analgesics for fever, teething and colicky pain.

“The nurses sell …… If they ask you to buy sometime and you say you don’t have money, they will say you are not concerned about your baby’s health” (Mother age 28 years).

Unemployed and single mothers complained of lack of money for transportation, and to buy new apparels/clothing to look socially acceptable at the clinic. A single mother aged 21 years with a seven-month old infant epitomized this by saying: “the day I have money, I come”. In few cases, mothers accused the community volunteers of demanding payment for service provision.

“Some of the workers (community volunteers) ask us to pay money. But I know that weighing is free of charge. I didn’t pay any money for my previous children. If you don’t pay, they will refuse to give you your child’s weighing card. That’s why I don’t like coming here” (Mother age 33 years).

The community volunteers admitted this allegation but attributed it to irregular remuneration by the District Health Directorate.

“The district is supposed to pay us every month. But we are hardly remembered. That is why we resolved to charge one Ghana cedi per client ….... yes, it is compulsory for them to pay” (Community Volunteer).

#### Implications of long waiting times

The mothers complained of undue delays and late commencement of activities at the clinic. This situation they bemoaned restricted them from trading on clinic days leading to loss of income. Another opportunity cost cited was inability to perform household responsibilities. Thirty-six and 29-year old mothers confirmed this concern:

“Anytime I bring my child for weighing, I’m not able to go and sell my wares”. I spend most part of the day at weighing”. “When my children return from school, there’s no one at home to take care of them. They just keep loitering until I return”.

The service providers on the other hand argued that the siting of clinics within wide catchment zones contributed to overcrowding at the sessions and stagnated workflow. Nonetheless, they reiterated that inadequate personnel and heavy work schedules were the primary reasons for the delays and not just the limited availability and accessibility to the service.

“We hear mothers complain about spending long hours at the clinic. But while most of these centres are understaffed, how can the work move fast? ........ Sometimes, the community health nurses have to pick-up vaccines at the directorate in the morning of the clinic. Unfortunately, we lack enough vehicles to convey them (the nurses) to their respective clinics, so they get there late”. (Public Health Nurse).

#### Unprofessional behaviours and attitudes

A major concern was inflexibility in scheduling appointments. The mothers believed their non-involvement in deciding on the visit date added to the dwindling attendance. Also, they expressed concern about the unprofessional behaviours exhibited by some staff which demoralized them from being consistent with schedules. Behaviours such as public scolding, lack of rapport, impatience to listen and lack of confidentiality especially during counselling sessions were specified. These communication lapses resulted in difficulty understanding the counselling messages. In instances of inadequate child growth, mothers lamented about inability to reach clear consensus on agreed actions to implement at home.

“Some of the nurses are unfriendly. If your child is not growing well, they will shout at you as if it is your fault. They won’t even allow you to explain yourself”. (29-year mother) .

#### High priority on immunization

The health workers seemingly focused on meeting immunization targets to the neglect of other vital components of the programme such as growth assessment and counselling on infant and young child feeding. Optimizing the use of the vaccines was vital. They clarified that once a vial of vaccine was opened; it ought to be used within a limited time. Low turn-up therefore implied vaccine wastage. However, they thought that the lack of knowledge by caregivers and their families on the usefulness of GMP played a role. On the other hand, the mothers patronized the child welfare services primarily because of the immunizations given to the infants, without which they were reluctant to attend.

“The role of immunization in reducing childhood illnesses has contributed to mothers not attending the sessions as most of them have never seen a child with poliomyelitis, serious measles, whooping cough, and so on before. Hence they do not believe these diseases are real and therefore have to vaccinate against it”. (Disease Control Officer)

“For most mothers, their main reason for sending their children to child welfare clinics is to receive vaccinations. When reports are collated, we notice that attendance is highest among participants that receive immunizations. This is making our surveillance on growth faltering difficult”. (Nutrition Officer)

“I just ignore coming if I know my baby is not due for injection…. because they only weigh the baby and give you the next date to come…. nothing more” (Mother age 35 years).

#### Misconceptions about the programme

According to the health workers, sections of the public held some misconceptions that prevented them from optimally utilizing the service. Allegations such as hoarding and using items meant for mother-infant pairs, extortions of money from clients, and making unwarranted financial gains through sale of items were mentioned.

“As was done in the past, some mothers expect to be given tokens as such weanimix, weighing pants and medicines free of charge. We need to tell them (mothers) that the situation has changed”. (Community Health Nurse)

### Integration of mixed methods results

Presented in Table 5 are key findings obtained from the quantitative and qualitative studies and its implications for preventive child health practice.

**Table 5:** Side-by-side display of key quantitative and qualitative findings and implications for practice

Attribute	Quantitative findings	Qualitative findings	Implications
Attendance to the growth monitoring and promotion programme	- Mean annual attendance 6.0 ± 2.9 - Proportion meeting ≥6 visits: 46.8% - Proportion meeting the recommended ≥9 visits: 13.6% - Overall drop-out rate: 59.5%	Attendance based on maternal age, parity, postpartum socio-cultural practices, financial constraints, irregular staff remuneration, delays, unprofessional staff behaviours, high premium on vaccinations & general misconceptions about GMP programme	Increase home visitations and target the following mothers: teenagers, single parents, women above 40 years, and those with parity above four children
Change in weight-for-age z-score during participation and the determining factors	WAZ of 69.1% of the children improved. Determinants: - Underweight at baseline (AOR:11.1, 95% CI:4.0–31.0) - ≥6 annual visits (AOR:2.2, 95% CI:1.1–4.1) ≥9 annual visits (AOR:4.7, 95% CI:1.4–15.1)	Deterioration in growth attributed to drop-out rates from the GMP programme, inadequate counselling, ineffective staff-client rapport, communication lapses, emphasis on achieving meeting vaccinations to the neglect of the other components of the programme	- Sensitization on contribution of routine growth monitoring and promotion to early child development and the dangers associated with unidentified growth faltering
Motivation to attend and level of satisfaction with service delivery	- Motivators for attendance were knowledge of child’s growth status and child vaccination. - 31% (95% CI: 25–37) of mothers satisfied - 59% (95% CI: 52–65) of mothers dissatisfied with service delivery	Satisfied as result of awareness of child’s growth and education provided on child care. Dissatisfaction resulted from: long waiting times; late start of clinic; uncomfortable clinic area, monies collected as services charges and negative staff attitude	- Primary healthcare systems should be strengthened to improve service delivery by increasing availability and accessibility to the service; staff supervision, training and monitoring

## Discussion

The aim of the quantitative study was to evaluate the trends in utilization of facility-based and community-based preventive child welfare services, change in weight-for-age z-scores of participants and caregivers’ level of satisfaction with the services received. Findings informed a qualitative follow-up study to explore viewpoints of mothers and health workers on why attendance to the clinics was below targets.

Average annual visit was six with overall drop-out rate of 60%. Reports from the Family Health Division of the Ghana Health Service shows that although children age 0–11 months continue to record the highest registration, trends in CWC service utilization has been sub-optimum compared to the 12–23 months and 24–59 months age groups [26]. The target for children age 0–11 months achieving nine visits per year has never been realized at the national level. For instance, from 2013 to 2015, average annual visits were 4.9, 5.7 and 5.5 respectively [26]. These number of annual visit are similar to the 5.95 and 6.05 mean annual attendance recorded in the community- and facility-based clinics. It is therefore not surprising that only 13.6% children met the nine-visit target which is an improvement over the national trend.

The drop-out rate of 59.5% is lower compared to the 74.1% observed in the Lusaka district of Zambia after a 3 month prospective follow-up [9]. Cross-sectional surveys conducted in both rural and urban Ghana show that on monthly basis, 30% of caregivers miss CWC sessions [18, 27]. These findings concur to our closed-cohort retrospective follow-up using secondary data. We observed that in the first month of the follow-up, clients who missed the sessions were about 30%.

The Ghana Health Service has linked drops in attendance to completion of majority of immunization and supplementations schedules. The health workers we interviewed acknowledged this situation. Widespread investments towards meeting immunization targets in Ghana is also accountable [28]. From the exit interviews, caregivers mentioned knowledge of child’s growth status as the most beneficial service received. But from the in-depth interviews, vaccinations ranked highest as the most important motivator for meeting schedules. Ashworth has observed that in preventive programmes, provision of medications including vaccinations are more valued than preventive services such as nutrition counselling [4].

Aside socio-demographic factors [29], long waiting times [30] transportation difficulties, inconvenient scheduling dates [18], poor client-staff relationship [31], and poorly motivated service providers [9] have been identified to reduce child welfare service utilization. However, other interesting findings emerged. These include public scolding of teenage and grand multiparous mothers, socio-cultural beliefs and practices that restricted early postpartum mothers-newborn pairs from going to public places and paying for an otherwise free service.

Utilization of preventive and promotion child health services in most developing countries vary widely depending on the mode of operation. Utilization of community-based programmes is usually higher than the traditional facility-based clinics. For example, in Honduras, monthly attendance rate among caregivers participating in the community-based growth promotion programme was significantly higher compared to those enrolled in the traditional health facility-based programme (60% vs. 40%) [32]. Average attendance in the Ugandan community-based programme was even higher (72%) [33]. In Rwanda, attendance was 53% in 2004 and increased to 78% in 2008 [34]. In our study however, caregiver-child pairs enrolled in the facility-based clinics tended to utilize the service more often.

Reasons for higher attendance to the facility-based clinics are evident from both the quantitative and qualitative studies. The community health nurses who run the facility-based clinics conduct more home visitation than their volunteer counterparts in the community-based clinics. In Ghana, home visitation is an effective mechanism to follow-up on defaulters, reinforce educational and counselling messages, observe practices in the natural environment, provide support, and link families to primary healthcare systems. It emerged from the in-depth interviews that due to infrequent remuneration, the free GMP programme was delivered at a fee by the volunteers, thereby deterring mothers from attending regularly. Meanwhile, frequent health worker-child contact is associated with better health outcomes [4]. This could explain why the proportion of underweight children increased among the community-based participants during the 12-months period. Despite this, more community-based clients were satisfied with the quality of services provided. Compliant of unprofessional behaviours were mainly against the community health nurses who run the facility-based clinics. This could contribute to the higher satisfaction expressed by the community-based clinic users. But this 31% satisfaction level is low compared to the 88% of primary healthcare users who expressed satisfaction in urban Nigeria [35].

Although WAZ of 30% participants deteriorated during the 12-months period, in Zambia, WAZ of all children (*n* = 698) deteriorated [9]. We found that more CWC visits and being underweight at enrolment were associated with improved WAZ. Unfortunately, children at high risk of malnutrition tend to attend CWC less often [13]. As noted from the qualitative study, the practice of scolding mothers whose children were faltering growth demotivated them from using the service.

### Strengths and limitations

This study has affirmed the potential effects of routine growth monitoring on improving the nutritional status of regular attendees and underweight children. This study is unique because of the innovation of applying mixed methods design to child development in primary healthcare systems research in Ghana. Methodology-wise, the combination of diverse designs both in the quantitative (cross-sectional survey and retrospective study using secondary data) and qualitative (case study and content analysis designs) research has enriched the findings as it has provided a holistic synopsis of the extent of child welfare service utilization and the factors mitigate patronage. Also, blending the conventional and directed approach as part of the content analysis design harnessed the strength of this qualitative methodology. The researchers did not only gain direct information from the caregivers without imposing preconceived theoretical perspectives but also expanded existing literature on the phenomenon of CWC utilisation. Instead of underweight rates, change in WAZ was used to build the regression model. WAZ corrects for age and eliminates the effect of change in age over time. Therefore, marginal changes in body weight and WAZ during participation were detected.

Nevertheless, the investigators acknowledge that the study is limited to one district in Ghana. To establish cause and effect, a prospective study would have been ideal. It is possible that the association of number of CWC visits predicting change in WAZ may actually be due to other unobserved differences in the mothers. That could not be determined in this observational study. It is worth noting that secondary data is not devoid of problems such as incomplete and multiple entries. Finally, background of the field investigator as a registered nurse and GMP service user could influence interpretations made.

## Conclusion

This study has shown that higher contact is associated with improved growth. For growth monitoring and promotion programmes to achieve the needed impact, intensity of contact with service providers is crucial. Strategies to enhance contact such as home visitation should be targeted at groups of people identified from both the quantitative and qualitative studies to be prone to sporadic attendance. These include malnourished children; early postpartum mother-child pairs; teenage, single and grand multiparous mothers; and children who have completed their vaccinations. To increase adherence to appointment schedules, health workers could consider consulting with caregivers to agree on suitable appointment dates. Parents and families need to be sensitized on the role of growth monitoring in promoting child health even after completion of vaccinations. Since the complementary role of community volunteers in increasing availability and accessibility of GMP services is indispensable, [36], issues relating to their remuneration require critical review by policy makers.

## Abbreviations

AOR: Adjusted odds ratio
CB: Community-based
CHN: Community health nurses
CI: Confidence interval
CWC: Child welfare clinic
FB: Facility-based
GMP: Growth monitoring and promotion
SD: Standard deviation
WAZ: Weight-for-age z-scores

## References

[1] Shekar M, Kakietek J, D’Alimonte M, Walters D, Rogers H, Eberwein J (2016). Investing in nutrition: the Foundation for Development: an investment framework to reach the global nutrition targets.

[2] Bhutta ZA, Das JK, Rizvi A, Gaffey MF, Walker N, Horton S (2013). Evidence-based interventions for improvement of maternal and child nutrition: what can be done and at what cost?. Lancet.

[3] WHO (2016). Monitoring of the achievement of the health-related Millennium Development Goals. Report by the Secretariat.

[4] Ashworth A, Shrimpton R, Jamil K (2008). Growth monitoring and promotion: review of evidence of impact. Matern Child Nutr..

[5] Daelmans B, Black MM, Lombardi J, Lucas J, Richter L, Silver K (2015). Effective interventions and strategies for improving early child development. Br Med J.

[6] Griffiths M, Del Rosso J (2007). Growth monitoring and the promotion of healthy young child growth. Evidence of effectiveness and potential to prevent malnutrition.

[7] Agbozo F, Colecraft E, Ellahi B (2016). Impact of type of child growth intervention program on caregivers’ child feeding knowledge and practices: a comparative study in Ga west municipality, Ghana. Food Sci Nutr.

[8] Ghana Health Service (2008). Nutrition and Malaria Control for Child Survival Project. Sub-project Manual.

[9] Charlton K, Kawana B, Hendricks M (2009). An assessment of the effectiveness of growth monitoring and promotion practices in the Lusaka district of Zambia. Nutrition.

[10] Nyavani SM, Xikombiso GM, Fhumudzani ML. Caregivers' interpretation of the growth chart and feeding practices of children under five years: a case of Greater Tzaneen municipality, South Africa. Journal of food and Nutr Res. 2016;4(6):369–76. http://pubs.sciepub.com/jfnr/4/6/5

[11] Roberfroid D, Pelto GH, Kolsteren P (2007). Plot and see! Maternal comprehension of growth charts worldwide. Tropical Med Int Health.

[12] de Onis M, Wijnhoven TM, Onyango AW (2004). Worldwide practices in child growth monitoring. J Pediatr.

[13] Ashworth A, Shrimpton R, Jamil K (2008). Growth monitoring and promotion:review of evidence of impact. Matern Child Nutr.

[14] Dewey KG, Adu-Afarwuah S (2008). Systematic review of the efficacy and effectiveness of complementary feeding interventions in developing countries. Maternal Child Nutr.

[15] Bilal SM, Moser A, Blanco R, Spigt M, Dinant GJ (2014). Practices and challenges of growth monitoring and promotion in Ethiopia: a qualitative study. J Health Popul Nutr.

[16] Owusu WB, Lartey A. Growth monitoring: experience from Ghana education. 1992;48:23.0.

[17] Bosu WK, Ahelegbe D, Edum-Fotwe E, Kobina AB, Kobina Turkson P (1997). Factors influencing attendance to immunization sessions for children in a rural district of Ghana. Acta Trop.

[18] Laar M, Marquis G, Lartey A, Gray-Donald K. Growth Monitoring and Promotion in rural Ghana: lack of motivation or tools? FASEB J. 2015;29(1Suppl):31–4. https://www.fasebj.org/doi/abs/10.1096/fasebj.29.1_supplement.31.4.

[19] Ghana Health Service (2014). 2014 Family Health Annu Rep.

[20] Creswell JW (2009). Research design: Qualitative, quantitative, and mixed methods approaches.

[21] Cohen J (1988). Statistical power analysis for the behavioral sciences.

[22] Whitley E, Ball J (2002). Statistics review 4: sample size calculations. Crit Care.

[23] Stake R. The art of case study research. Thousand Oaks: SAGE Publications Inc; 1995.

[24] Hsieh H-F, Shannon SE (2005). Three approaches to qualitative content analysis. Qual Health Res.

[25] Fetters MD, Curry LA, Creswell JW (2013). Achieving integration in mixed methods designs—principles and practices. Health Serv Res.

[26] Ghana Health Service (2015). Family health Annu Rep.

[27] Gyampoh S, Otoo GE, Aryeetey RNO (2014). Child feeding knowledge and practices among women participating in growth monitoring and promotion in Accra, Ghana. BMC Pregnancy and Childbirth.

[28] LaFond A, Kanagat N, Steinglass R, Fields R, Sequeira J, Mookherji S. Drivers of routine immunization coverage improvement in Africa: findings from district-level case studies. Health policy and planning. 2014;30(3):298-308. 10.1093/heapol/czu011. 10.1093/heapol/czu011 PMC4353894 24615431

[29] Cameron E, Heath G, Redwood S, Greenfield S, Cummins C, Kelly D, et al. Health care professionals’ views of paediatric outpatient non-attendance: implications for general practice. Fam Pract. 2014;31(1):111-17. 10.1093/fampra/cmt063. 10.1093/fampra/cmt063 PMC3902212 24243869

[30] Turkson P. Perceived quality of healthcare delivery in a rural district of Ghana. Ghana Med J. 2009;43(2):65-70. 10.4314/gmj.v43i2.55315 PMC3039236 21326844

[31] Martin C, Perfect T, Mantle G (2005). Non-attendance in primary care: the views of patients and practices on its causes, impact and solutions. Fam Pract.

[32] Van Roekel K, Plowman B, Griffiths M, Vivas V, de Alvarado J, Matute M (2002). BASICS II Midterm evaluation of the AIN program in Honduras.

[33] Muyeti RS, Miller del Rosso J. Uganda Community Based Growth promotion: Program review. Uganda. In: Uganda program for human and holistic development (UPHOLD), The Manoff group and USAID: JSI Res Training Inst Inc, 2008; 2007.

[34] Ngirabega J, Leonard W, Munyanshongore C, Dramaix-Wilmet M (2010). Utilization of community based growth monitoring services by eligible children in rural Rwanda. Rwanda Medical Journal.

[35] Sule S, Olawuyi O, Afolabi O, Onajole A, Ogunowo B (2013). Caregivers knowledge and utilization of child health services in an Urban District of Lagos, Nigeria. West African J Med.

[36] Afulani PA, Awoonor-Williams JK, Opoku EC, Asunka J (2012). Using community health workers in community-based growth promotion: what stakeholders think. Health Educ Res.

